# Replacement in angiogenesis research: Studying mechanisms of blood vessel development by animal-free *in vitro*, *in vivo* and *in silico* approaches

**DOI:** 10.3389/fphys.2022.981161

**Published:** 2022-08-17

**Authors:** Matthias W. Laschke, Yuan Gu, Michael D. Menger

**Affiliations:** Institute for Clinical and Experimental Surgery, Saarland University, Homburg, Germany

**Keywords:** angiogenesis, endothelial cells, migration, microfluidics, zebrafish, proliferation, mathematical modeling, spheroid

## Abstract

Angiogenesis, the development of new blood vessels from pre-existing ones, is an essential process determining numerous physiological and pathological conditions. Accordingly, there is a high demand for research approaches allowing the investigation of angiogenic mechanisms and the assessment of pro- and anti-angiogenic therapeutics. The present review provides a selective overview and critical discussion of such approaches, which, in line with the 3R principle, all share the common feature that they are not based on animal experiments. They include *in vitro* assays to study the viability, proliferation, migration, tube formation and sprouting activity of endothelial cells in two- and three-dimensional environments, the degradation of extracellular matrix compounds as well as the impact of hemodynamic forces on blood vessel formation. These assays can be complemented by *in vivo* analyses of microvascular network formation in the chorioallantoic membrane assay and early stages of zebrafish larvae. In addition, the combination of experimental data and physical laws enables the mathematical modeling of tissue-specific vascularization, blood flow patterns, interstitial fluid flow as well as oxygen, nutrient and drug distribution. All these animal-free approaches markedly contribute to an improved understanding of fundamental biological mechanisms underlying angiogenesis. Hence, they do not only represent essential tools in basic science but also in early stages of drug development. Moreover, their advancement bears the great potential to analyze angiogenesis in all its complexity and, thus, to make animal experiments superfluous in the future.

## Introduction

Angiogenesis is a fundamental biological process defined as the development of new blood vessels from pre-existing ones (Ribatti and Pezzella, 2021). Because cell survival and proliferation are crucially dependent on a sufficient oxygen and nutrient supply, angiogenesis is a major prerequisite for tissue formation and growth. Accordingly, blood vessel formation plays an essential role during embryogenesis and wound healing (Fajersztajn and Veras, 2017; Sorg et al., 2018). Moreover, it is important for the physiological reproductive function of the placenta, ovary and uterus (Reynolds et al., 1992; Vollmar et al., 2001; Laschke et al., 2008). On the other hand, many pathological conditions are typically driven by angiogenesis, such as tumor growth and metastasis (Folkman, 2002), endometriosis (Laschke and Menger, 2018), rheumatoid arthritis (Wang Y. et al., 2021), ocular neovascular diseases (Plastino et al., 2021) and chronic inflammatory skin disorders (Lee et al., 2021). Hence, there is a strong interest in uncovering molecular and cellular angiogenic mechanisms and in assessing the pro- and anti-angiogenic effects of various agents to provide the basis for the establishment of novel therapeutic approaches. For this purpose, a broad spectrum of *in vitro*, *in vivo* and *in silico* assays and models has been introduced in angiogenesis research during the last decades (Nowak-Sliwinska et al., 2018).

There is no doubt that animal studies markedly contribute to a better understanding of angiogenesis under different physiological and pathological conditions. However, they also raise major ethical concerns, because they face the fundamental conflict of interest between the claim of advancing scientific knowledge and the protection of animals. To address this critical issue, William Russell and Rex Burch introduced the so-called 3R principle in 1959, which defines central criteria to perform animal experiments in a more humane way (Russell and Burch, 1959). This principle is based on the 3Rs “replacement, reduction and refinement”. Replacement means that animal models should be replaced by animal-free approaches whenever possible. If this is not completely achievable, researchers should at least reduce the number of individual animals required to generate statistically valid and reproducible data to an absolute minimum. Refinement, in turn, focuses on any decrease in the incidence or severity of inhumane procedures applied to those animals, which are still to be used (Russell and Burch, 1959).

**FIGURE 1:**
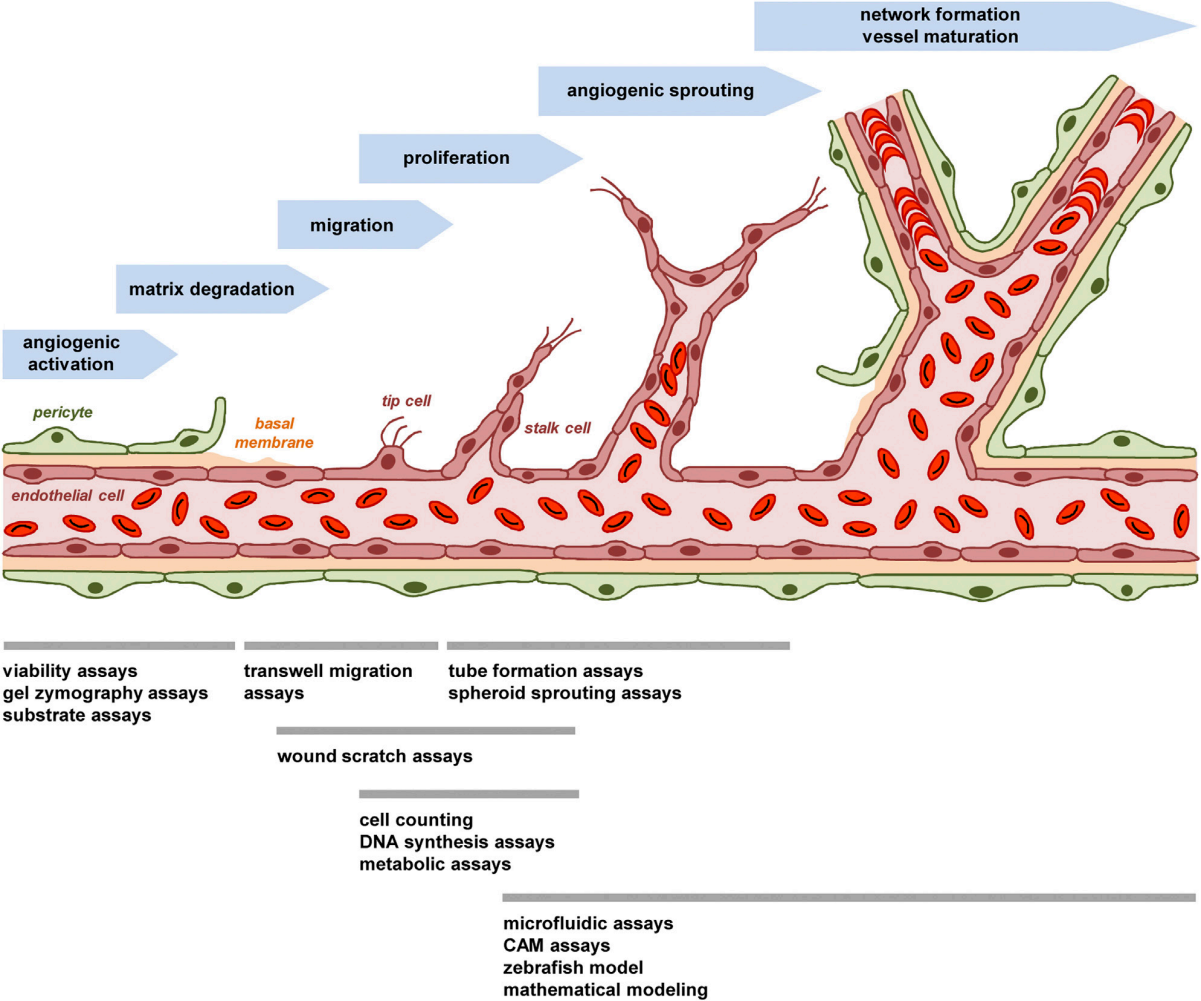
The process of angiogenesis and animal-free approaches for its investigation. The process of angiogenesis can be subdivided in several well-characterized steps, which involve i) the angiogenic activation of microvessels by growth factors, ii) the detachment of stabilizing pericytes and the degradation of the basal membrane by MMPs, iii) the migration of endothelial tip cells towards an angiogenic stimulus and iv) the proliferation of following endothelial stalk cells, which results in v) the formation of angiogenic sprouts. These sprouts develop a lumen and vi) finally interconnect with each other to new blood-perfused microvascular networks, which are stabilized by the formation of a new basement membrane and the recruitment of perivascular cells. To study this process *in vitro*, *in vivo* or *in silico*, multiple animal-free angiogenesis assays and models are available focusing on different steps of blood vessel formation.

As from an ethical point of view replacement is the most desirable aim, the present review article selectively provides an overview of common animal-free approaches in angiogenesis research. These approaches allow for the analysis of multiple biological mechanisms that are of utmost importance for the development of new blood vessels (Figure 1). Hence, they do not only represent essential tools in basic science but also in early stages of drug development.

## The dynamic process of angiogenesis

A major prerequisite for the adequate use and interpretation of angiogenesis assays is the basic understanding of the dynamic process of blood vessel formation, which is characterized by the coordinated interaction of humoral factors and different cell types. It is initiated by a local imbalance of pro-angiogenic factors, such as vascular endothelial growth factor (VEGF) and basic fibroblast growth factor (bFGF), and anti-angiogenic factors, such as endostatin and thrombospondin (Naumov et al., 2006; Hu et al., 2014). Potent triggers for this so-called angiogenic switch are tissue hypoxia and inflammation (Hashimoto and Shibasaki, 2015; Ridiandries et al., 2016). This results in the detachment of stabilizing pericytes from the wall of pre-existing (parent) microvessels and the angiogenic activation of microvascular endothelial cells, which start to degrade their basement membrane by the release of proteolytic matrix metalloproteinases (MMPs) (Carmeliet and Jain, 2011). In a next step, the endothelial cells migrate out of the vessel wall. Tightly regulated by Notch/DLL4 signaling, they differentiate into filopodia-forming tip cells, which spearhead new vessel sprouts towards an angiogenic stimulus and are followed by proliferating stalk cells (Potente et al., 2011). The elongating sprouts develop a lumen and interconnect with each other to blood-perfused microvascular networks. These networks are finally stabilized by the formation of a new basement membrane and the recruitment of perivascular cells (Sun et al., 2015).

This brief description indicates that the process of blood vessel development can be subdivided in several well-characterized steps, which involve angiogenic activation, matrix degradation, endothelial cell migration and proliferation as well as sprouting, network formation and vessel maturation (Figure 1). Comparably, most animal-free angiogenesis assays can also be subdivided according to their main mechanism(s) of action to be studied (Figure 1).

## *In vitro* assays

### Endothelial cells

Endothelial cells are the primary target cells of *in vitro* angiogenesis assays. When performing such assays, the choice of the right endothelial cell type already represents the first major challenge. Immortalized cell lines may not be recommended, because they exhibit altered growth control and survival mechanisms (van Beijnum et al., 2008). Hence, primary endothelial cells are commonly used. They are available from different species, such as mice, pigs, cattle and humans. For the sake of generating translational results human cells should be preferred if possible. These can be harvested from large vessels, such as human arterial endothelial cells (HAECs) and human umbilical vein endothelial cells (HUVECs). Particularly the latter ones are widely used in angiogenesis studies due to their availability, low costs of maintenance and capability of forming capillary structures (Stryker et al., 2019). However, angiogenesis typically occurs within the microvasculature and it is well known that endothelial cells from macro- and microvessels markedly differ in terms of their structural and functional phenotype (King et al., 2004; Hewett, 2016). To address this issue, human microvascular endothelial cells may alternatively be used. There are different sources for these cells. For instance, commercially available human dermal microvascular endothelial cells (HDMECs) are isolated from the dermis of juvenile foreskin and adult skin. It should be considered that they comprise a mixture of blood and lymphatic endothelial cells (Rossi et al., 2010). Moreover, it should be noted that endothelial cells even exhibit heterogenous phenotypes within the microvasculature of distinct tissues and they may change their behavior in angiogenesis assays when stored for multiple passages (Stryker et al., 2019; Hennigs et al., 2021). The latter problem can be overcome by establishing a master cell bank with cells in the identical early passage and working cell banks for daily experiments.

### Endothelial cell viability assays

The assessment of endothelial cell viability is often the initial step in studies evaluating potential pro- or anti-angiogenic effects of test compounds (Laschke et al., 2011; Gu et al., 2013). Indeed, it is important to first identify non-cytotoxic dosages of such compounds, which can then be applied for the treatment of viable endothelial cells in subsequent angiogenesis assays. For this purpose, several cytotoxicity assays exist, which are based on the fact that dead cells lose their membrane integrity, allowing the movement of otherwise non-permeable molecules into or out of the cells (Riss et al., 2019).

Frequently used dyes that selectively penetrate damaged cell membranes and, thus, stain dead cells are trypan blue or the fluorescent DNA binding dye propidium iodide (Rusovici et al., 2011). The number of stained cells can be analyzed by means of a hematocytometer or automated cell counters. However, when using this approach, it should be considered that the dyes themselves can be toxic to the analyzed cell population dependent on the duration of exposure and the susceptibility of the used cells.

A commonly detected enzyme, which leaks from the cytoplasm of dead cells into the culture medium, is lactate dehydrogenase (LDH) (Becker et al., 2021). LDH catalyzes the conversion of pyruvate to lactate, which is associated with the conversion of NAD^+^ to NADH. The activity of NADH is finally used to reduce specific substrates in the culture medium into colored, fluorescent or luminogenic products for detection purposes (Riss et al., 2019).

In general, the choice of an endothelial cell viability assay depends on various factors, such as the experimental setting and duration, the desired throughput, the costs of required reagents or the available technical laboratory equipment. It is always advisable to confirm the results from one assay by another one or by combining different cytotoxicity markers. Moreover, it is important to include adequate positive and negative controls.

### Matrix degradation

Upon angiogenic activation, endothelial cells of pre-existing vessels release different types of proteases, particularly MMPs, which are necessary for the degradation of the vessels’ basement membrane and the surrounding extracellular matrix (ECM) (Quintero-Fabián et al., 2019). The activity of MMPs can be analyzed by means of gel zymography assays (Wilkesman and Kurz, 2012). For this purpose, protease-containing lysates or supernatants from endothelial cells are electrophoresed through SDS-PAGE gels, which consist of MMP substrates, such as collagen or gelatin, and polyacrylamide. After stopping the proteolytic process, these gels are stained with Coomassie Blue to identify transparent, hydrolized areas in negative contrast as correlates for the relative amount of present proteases (Lombard et al., 2005; Heo et al., 2020). This inexpensive approach can be used to detect qualitatively or semi-quantitatively the relative levels of specific MMPs, but it should be noted that it is time-consuming and not suitable for high-throughput screening.

Alternatively, it is possible to perform substrate assays by incubating endothelial cells or their supernatants with MMP substrates that are fluorescently labeled, biotinylated or succinylated for the measurement of their hydrolysis (Menges et al., 1997; Baragi et al., 2000; Ratnikov et al., 2000). This allows rapid, quantitative and large-scale screening experiments of potential MMP stimulators and inhibitors (Goodwin, 2007). However, it is difficult to differentiate between the activity of individual MMPs, because multiple MMPs may contribute to the degradation of a single substrate. To overcome this problem, additional steps can be included, such as the isolation of distinct MMPs by means of specific antibodies (Hawkins et al., 2013).

### Endothelial cell migration assays

**FIGURE 2:**
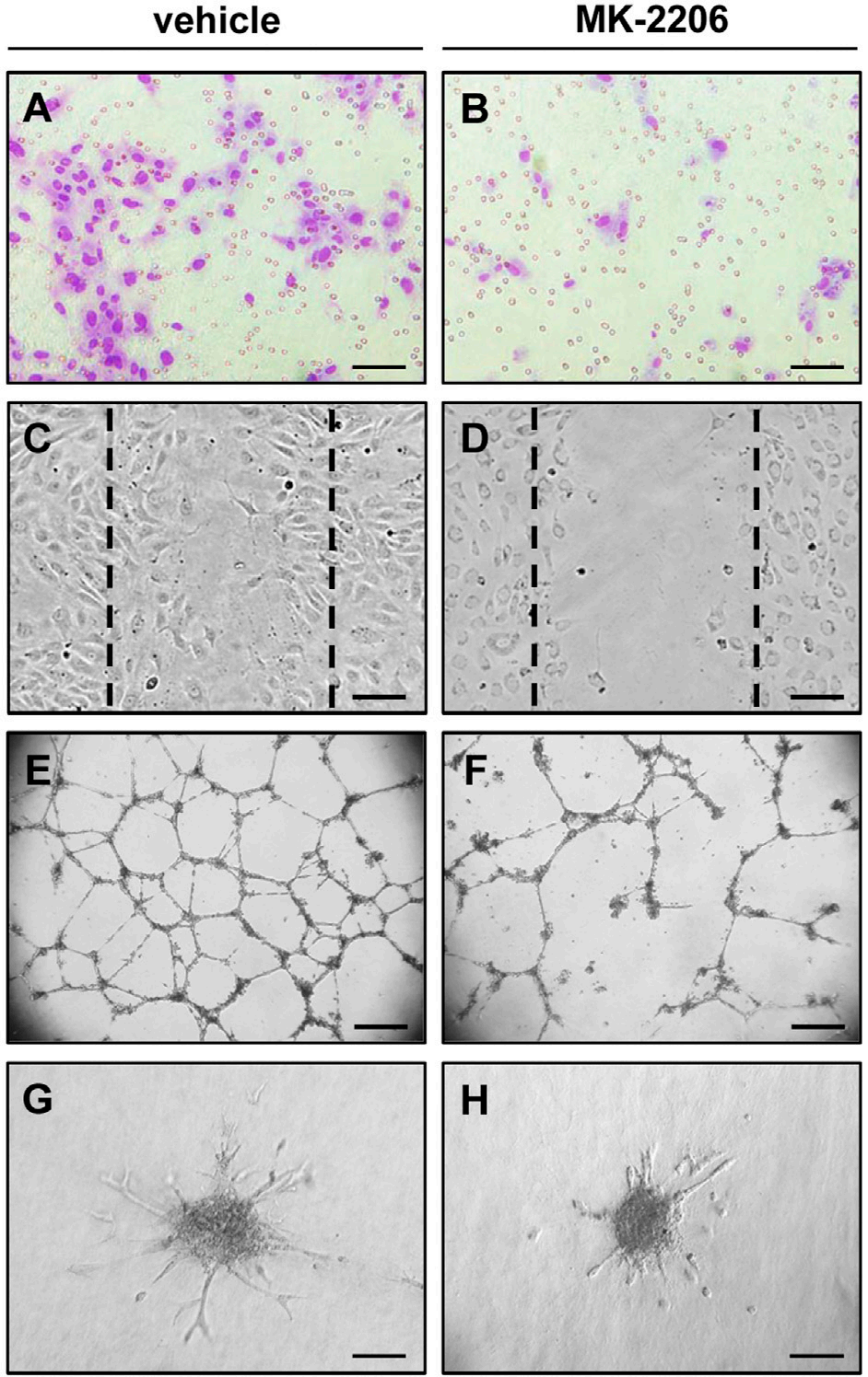
*In vitro* angiogenesis assays indicating the anti-angiogenic activity of the AKT inhibitor MK-2206. **(A,B)** Transwell migration assay: Microscopic images of HUVECs after their migration through the 8 µm pores of the polycarbonate filters of a transwell migration assay to the lower membrane surface. The cells were pre-cultivated for 24 h with vehicle **(A)** or 10 µM MK-2206 **(B)** and then analyzed in the transwell migration assay for additional 5 h. Scale bars: 80 µm. **(C,D)** Wound scratch assay: Microscopic images of HUVEC monolayers, which were exposed for 18 h to vehicle **(C)** or 10 µM MK-2206 **(D)**, and then scratched. Images of the scratched wounds were taken after 6 h. The initial scratch borders are marked with broken lines. Scale bars: 160 µm. **(E,F)** Tube formation assay: Microscopic images of tube-forming HUVECs on Matrigel, which were exposed for 18 h to vehicle **(E)** or 10 µM MK-2206 **(B)**. Scale bars: 450 µm. **(G,H)** Spheroid sprouting assay: Microscopic images of sprouting HUVEC spheroids, which were treated for 24 h with vehicle **(G)** or 10 µM MK-2206 **(H)**. Scale bars: 140 μm.

The migration of endothelial cells towards a chemotactic stimulus crucially determines angiogenic sprout formation. Accordingly, angiogenesis studies usually include endothelial cell migration assays. The most frequently used assays are transwell migration assays and wound scratch assays (Figure 2).

Transwell migration assays are based on the principle of the Boyden chamber assay, which has originally been established by Steven Boyden in 1962 to monitor the chemotaxis of immune cells (Boyden, 1962). Nowadays, many versions of these assays are commercially available. They all share the common design of a chamber or well with an upper and a lower medium-filled compartment, which are separated from each other by a filter membrane with pores of 3–8 µm in diameter. The lower compartment contains chemotactic factors or factor-producing cells (Hilkens et al., 2014; Wang X. et al., 2021). Hence, when the upper compartment is loaded with endothelial cells, they migrate through the porous filter membrane towards the lower compartment. After a few hours, the membrane is harvested and the migrated endothelial cells at its bottom side are stained to count them (Gu et al., 2016). Of interest, this rather simple approach can be further modified to mimic the invasion of endothelial cells into ECM compounds, for which the endothelial secretion of MMPs is necessary. For this purpose, the filter membrane is coated with collagen, fibronectin or Matrigel (Albini and Benelli, 2007; Xu et al., 2019). While transwell migration assays can be rapidly performed in less than 24 h, it should be considered that the required filter membranes are rather expensive. Moreover, these assays are crucially dependent on a chemical gradient, which may equilibrate throughout the chamber over longer time periods. This can result in random motion instead of directed migration of the analyzed endothelial cells. Accordingly, pilot experiments are often required to define the ideal observation period for transwell migration assays depending on the used chemotactic stimulus as well as the used cell type and its physiological migratory capacity.

In contrast to transwell migration assays, wound scratch assays do not assess chemotaxis towards an angiogenic stimulus, but only undirected, lateral migration of endothelial cells in response to test compounds in the culture medium (Nowak-Sliwinska et al., 2018). For this purpose, the cells are first grown to a confluent monolayer in a culture dish. Subsequently, a “wound” is manually scratched into the monolayer by means of a pipette tip or a cell scraper (Gu et al., 2021). Because this may result in wounds of unequal size, it is also possible to put standardized silicon templates as placeholders in the culture dish prior to cell seeding, which are removed at the beginning of the experiment (Cappiello et al., 2018). The endothelial cells at the wound edges begin to migrate into the scratched area and progressively close the wound gap. This usually requires 2–4 days and quantitative assessment of wound closure over time can be analyzed by time-lapse microscopy or by microscopy at defined observation time points (Gu et al., 2017; Korybalska et al., 2017). It should be noted that this process is not solely dependent on the migration but also on the proliferative activity of endothelial cells. To exclude this bias, the cells can be exposed, additionally to the test compounds, to a proliferation inhibitor, such as mitomycin (Taniguchi et al., 2018). Meanwhile, several companies offer systems for the fully automated, high-throughput performance and analysis of wound scratch assays during large-scale screens (Nowak-Sliwinska et al., 2018). This indicates that this type of assay is well-established and broadly used, not only to investigate the migratory activity of endothelial cells, but also of various other benign and malignant cell types (Ideta et al., 2021; Wiegand et al., 2021; Fong et al., 2022).

### Endothelial cell proliferation assays

Proliferation can be analyzed by simply counting the number of cultivated endothelial cells by means of a hematocytometer to create growth curves (Lou et al., 1997). For standardized measurements, it is important that the cell density of the culture is carefully controlled, since loss of cell-cell contacts may markedly promote proliferation, whereas confluency may result in contact inhibition and, thus, a non-proliferative, quiescent state of the cells (Staton et al., 2009). Although this approach is straightforward and does not require expensive equipment, it is time-consuming and prone to sampling error. Alternatively, several automated platforms are meanwhile available, which allow high-throughput cell counting in microplates over time (Nowak-Sliwinska et al., 2018).

DNA synthesis assays for the assessment of cell proliferation take advantage of modified nucleotides, which are added to the culture medium and are incorporated into the newly synthesized DNA during the S phase of the cell cycle. For this purpose, the radioactive compound 3H-thymidine in combination with liquid scintillation counting has been classically used (Lou et al., 1997; Nishizuka et al., 2001). More recently, non-radioactive nucleotide analogs, such as 5-bromo-2′-deoxyuridine (BrdU) or 5-ethynyl-2′-deoxyuridine (EdU), offer more environmentally friendly alternatives, which can be detected by flow cytometry, enzyme-linked immunosorbent assay (ELISA) or immunohistochemistry (Becker et al., 2021; Hillenmayer et al., 2022; Zhang et al., 2022).

Other frequently used proliferation assays are based on the exposure of endothelial cells to tetrazolium salts, such as 3-(4,5-dimethylthiazol-2-yl)-2,5-diphenyltetrazolium bromide (MTT assay) or 2-(4-iodophenyl)-3-(4-nitrophenyl)-5-(2,4-disulfophenyl)-2H-tetrazolium (WST-1 assay) (Gu et al., 2013; Laranjeira et al., 2013; Amoorahim et al., 2020). These salts are converted by the cells’ mitochondrial dehydrogenase to purple formazan, which is then spectrophotometrically detected in the culture medium and correlates with the number of viable cells. However, this is a rather indirect approach to measure cell proliferation. In fact, it should be considered that the results of these so-called metabolic assays may be markedly biased by compounds that directly affect mitochondrial function or cell viability. Accordingly, it is highly recommended to combine these assays with other proliferation and viability assays for the generation of robust data sets on the proliferating activity of endothelial cells.

### Tube formation assays

Tube formation assays have been established to investigate the morphogenesis and differentiation of endothelial cells, which contribute to the formation of microvascular networks in later stages of angiogenesis. They are based on the observation that endothelial cells spontaneously develop interconnected tubular structures within a few hours, when they are seeded and cultivated on different types of ECM (Figure 2). These structures are considered as rudimentary capillary-like tubes with tight junctions between individual endothelial cells (Auerbach et al., 2003), which, under certain conditions, are also able to form a lumen as demonstrated by light and electron microscopy (Connolly et al., 2002). Of interest, this lumen formation is closely associated with apoptotic cell death outside and inside the tubular structures (Segura et al., 2002).

There are several issues, which need to be considered when setting up a tube formation assay. The seeding density of endothelial cells crucially determines their capacity of developing tubular meshes. In fact, too low seeding densities prevent tube formation, whereas too high densities can result in large, undifferentiated cell clusters (Staton et al., 2004). The kinetics and extent of endothelial tube formation is further determined by the used matrix (Zimrin et al., 1995). Nowadays, Matrigel is the most frequently used substrate for this assay. It contains angiogenic growth factors, ECM and basement membrane proteins from murine Engelbreth-Holm-Swarm sarcoma (Schuetz et al., 1988). Matrigel is also commercially available in a growth factor-reduced form, which may be particularly useful when investigating a potential pro-angiogenic activity of test compounds (Tan et al., 2010). However, it should be noted that besides endothelial cells also other cell types, such as fibroblasts and cancer cells, are able to develop tubular structures on Matrigel (Donovan et al., 2001). This complicates the interpretation of tube formation assays that use mixed cell populations. On the other hand, such a modified co-culture approach bears the opportunity to study the interaction of endothelial cells with pericytes during the process of tube formation (Schmitt et al., 2020).

Another interesting modification is the transfer of the classical two-dimensional (2D) tube formation assay to a three-dimensional (3D) level. This can be achieved by embedding endothelial cell-coated microspheres in a fibrin gel, also named fibrin bead assay (Nehls and Drenckhahn, 1995; Carpentier et al., 2020), or by cultivation of confluent endothelial cells in between different layers of ECM components (Gagnon et al., 2002).

2D and 3D tube formation assays allow the analysis of multiple parameters, such as the total tube length and mesh area or the total number of tubes, junctions and meshes. These parameters can be manually assessed, which is time-consuming, operator-dependent and not suitable for large-scale screenings. Alternatively, a variety of image analysis programs for an automated analysis are on the market (Boizeau et al., 2013; Carpentier et al., 2020). However, they should be used with caution, because they are usually based on thresholding to discriminate cells and tubes from background. Hence, the validity of such an automated analysis is always dependent on the quality of the used images.

### Spheroid sprouting assays

Spheroids are multi-cellular aggregates, which mimic the 3D environment with intensive cell-cell contacts of natural tissues. Accordingly, they react more physiologically to external stimuli and exhibit improved biological functions when compared to two-dimensional single cell systems (Laschke and Menger, 2017a). Spheroids of defined size and cell number can be rapidly fabricated in large amounts by various methods, including hanging drop and cell suspension cultures or the liquid overlay technique (Fonoudi et al., 2015; Neto et al., 2015; Costa et al., 2018). In 1998, Korff and Augustin introduced collagen-embedded endothelial cell spheroids as vascularization units in angiogenesis research (Korff and Augustin, 1998). The endothelial cells on the surface of these spheroids exhibit a quiescent phenotype (Korff and Augustin, 1998). However, cultivation in ECM activates the cells and stimulates the growth of angiogenic sprouts out of the spheroids (Figure 2), which can be quantitatively assessed over time.

During the last 2 decades, this approach has been diversely modified to serve as a versatile tool for the analysis of molecular and cellular determinants of blood vessel development, including different endothelial cell phenotypes, pro- and anti-angiogenic factors as well as cell-matrix interactions (Laschke and Menger, 2017b). Of interest, it also allows for the investigation of complex co-culture spheroids by combining endothelial cells with other cell types (Baal et al., 2009; Walser et al., 2013; Shah et al., 2019). Thus, it is possible to study angiogenesis in tissue-specific microenvironments. Given the fact that tumor cells behave different in co-culture with endothelial cells (Upreti et al., 2011), this can be advantageous for the screening of novel anti-angiogenic cancer drugs. Moreover, it can provide deeper insights into the regulation of blood vessel development by disease-related factors. For instance, Maracle et al. (2017) exposed co-culture spheroids composed of endothelial cells and fibroblast-like synoviocytes to rheumatoid arthritis synovial fluid to demonstrate that inflammatory synovial angiogenesis is primarily induced by NF-κB signaling and can be inhibited by the anti-angiogenic agent anginex.

### Microfluidic assays

**FIGURE 3:**
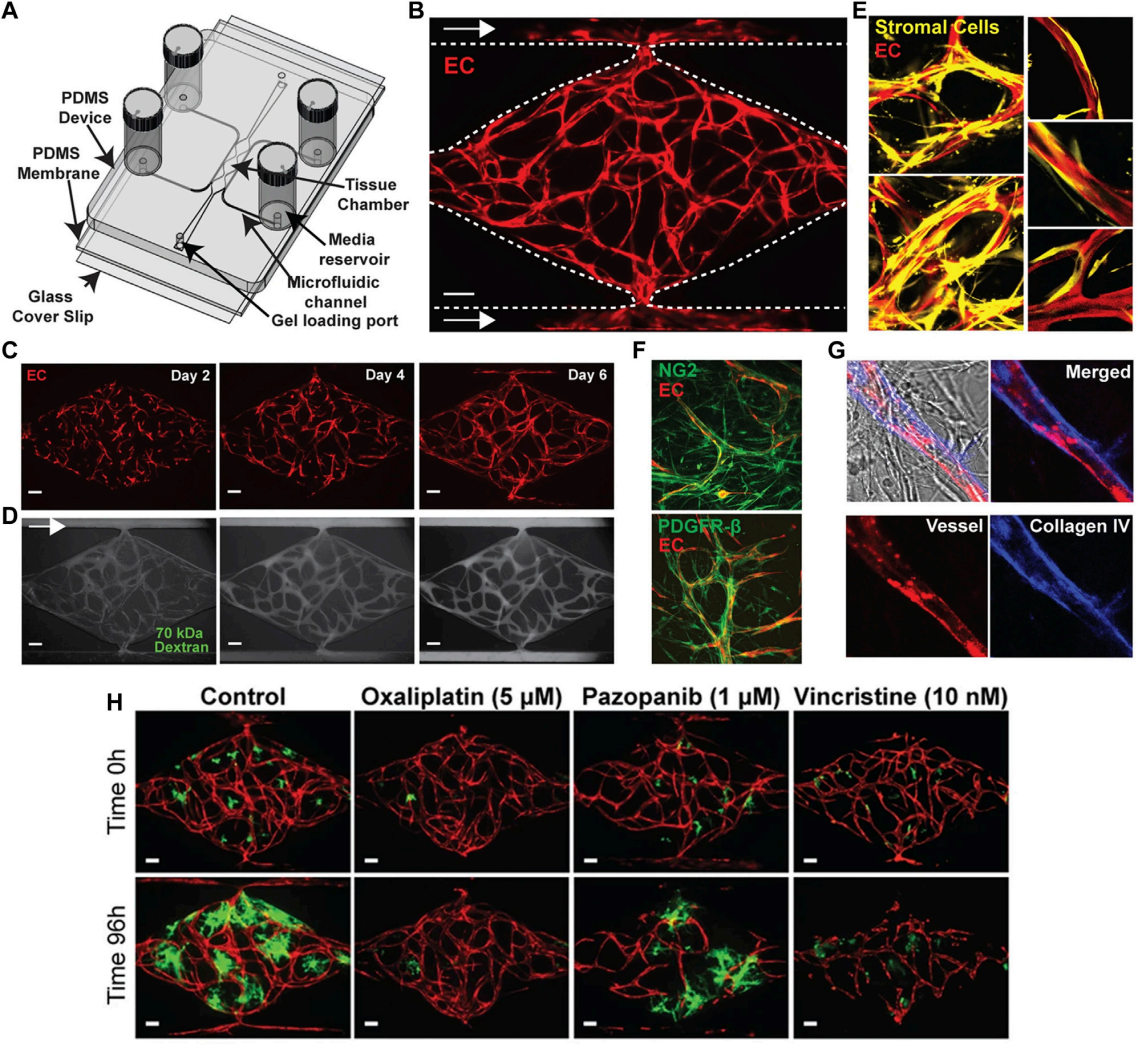
Microfluidic vascularized micro-organ platform for the analysis of tumor angiogenesis and the evaluation of anti-angiogenic compounds according to Sobrino et al. (2016). **(A)** Schematic of the microfluidic platform. The vascularized micro-organ platform consists of a thick layer of polydimethyl siloxane (PDMS) containing patterned tissue chambers, which can be loaded with endothelial cells, perivascular cells, tumor cells and ECM, and microfluidic channels, bonded to a thin PDMS membrane and a glass cover slip. Three tissue chambers at the center are connected to two adjacent channels by two capillary burst valves that retain the mixture of cells and ECM inside the chambers. At the two ends of the tissue chambers are two gel loading ports, through which the cell-ECM suspension is introduced. Four media reservoirs are attached to the inlets and outlets of the microfluidic channels. **(B)** Representative tissue chamber with a fully-developed vascular network on day 7. Lentivirally transduced endothelial cells (red) are visualized by confocal microscopy. Supporting stromal cells are unlabeled. Endothelial cells migrate outward and anastomose with the microfluidic channels. Scale bar: 100 μm. **(C)** Representative time course of vascular network development (day 2, 4 and 6). Scale bar: 100 μm. **(D)** Representative time course of 70 kDa fluorescein isothiocyanate-dextran perfusion through the vascular network on day 7. Inflow is top left and outflow bottom right. The vascular network is fully perfused within 15 min. Endothelial cells were labeled with mCherry. Scale bar: 100 μm. **(E)** Confocal imaging of lentivirally transduced endothelial cells (red) and stromal cells (yellow) reveals that stromal cells take up a perivascular position. High magnification views on the right. **(F)** Immunostaining for platelet-derived growth factor receptor (PDGFR)-β and nerve/glial antigen (NG)2 (both green). Endothelial cells are expressing mCherry. **(G)** Collagen IV staining (blue) identifies basement membrane deposition. **(H)** Anti-cancer drugs that target tumor, vasculature, or both. Lentivirally transduced colorectal cancer HCT116 cells (green) in a vascularized micro-organ platform exposed to Pazopanib (1 μM), Oxaliplatin (5 μM) and Vincristine (10 nM). Images before and after drug exposure are of the same vascularized micro-organ platform. Scale bar: 100 μm. Reproduced with permission from Nature under the terms of the Creative Commons Attribution 4.0 International License (http://creativecommons.org/licenses/by/4.0/).

During the last 15 years, the number of microfluidic angiogenesis studies has rapidly increased, because microfluidic assays ideally bridge the gap between the aforementioned approaches and the later on discussed *in vivo* models. In fact, they offer the unique possibility to investigate *in vitro* the dynamic process of blood vessel development with special attention to fluid mechanical stimuli, such as shear forces and interstitial flow, which are important determinants of angiogenic sprouting and network formation (Song and Munn, 2011; Winkelman et al., 2021). For this purpose, sophisticated microfabrication techniques have been established. They allow the generation of user-defined 2D and 3D microchannel networks and interconnected compartments to mimic complex *in vivo* environments that can be analyzed over weeks (Morgan et al., 2013; Sobrino et al., 2016; Akbari et al., 2017; Wang et al., 2017; Yue et al., 2021) (Figure 3). These systems provide easily accessible, standardized conditions with spatiotemporal control of (bio)chemical and physical stimuli (Nishimura et al., 2020). In basic angiogenesis research, they have been broadly used to study the impact of hemodynamic forces (Akbari et al., 2019; Arpino et al., 2021; Zhao et al., 2021) as well as cell-cell (Amemiya et al., 2021; Bai et al., 2021; Walji et al., 2021) and cell-matrix interactions (Wang W. Y. et al., 2021; Liu et al., 2021) on the mechanisms of blood vessel development. Moreover, they represent attractive tools for the high-throughput screening of anti-angiogenic drugs (Kim et al., 2015; Sobrino et al., 2016; Kim et al., 2021) (Figure 3). However, despite these advantages and the versatility of microfluidic assays, they are still not routinely used in most biology laboratories. This is due to the fact that microfluidic studies require special expertise and the appropriate equipment for microsystems engineering. To overcome this problem, it is necessary to establish more user-friendly microfluidic systems, which are commercially available and can be run without extensive technical training (Young, 2013). If this succeeds, these systems bear the potential to replace more classical *in vitro* approaches in angiogenesis research.

## *In vivo* models

At first glance, this section seems not to fit in the present review article focusing on animal-free approaches. However, the *in vivo* models described below share the common feature that they are exclusively based on primitive organisms, i.e., fertilized chicken eggs and zebrafish larvae. Due to the stage of their development, these organisms lack pain perception. This is also a major reason why by law in many countries these models do not have to be registered as animal experiments and are even recommended as suitable approaches for the reduction and replacement of such.

### Chick chorioallantois membrane assay

The chick chorioallantois membrane (CAM) assay is a well-established *in vivo* approach, which has been widely used in many different modifications and various areas of research for decades (Ribatti, 2017; Chu et al., 2022). It uses the CAM as target tissue for the analysis of angiogenic processes. The CAM forms by the fusion of the allantois and chorion in fertilized chicken eggs and serves as a transient gas exchange surface for the embryo. It rapidly develops between day 3 and 9 into a highly vascularized tissue layer containing a dense network of arterioles, capillaries and venules (Laschke et al., 2006). In the classic in ovo assay, a circular, sealable observation window is prepared into the eggshell for the repeated analysis of the CAM (Schmitd et al., 2019). Alternatively, ex ovo assays with shell-less embryo cultures have been introduced, which enable an easier access to larger areas of the CAM (Vargas et al., 2009; Merlos Rodrigo et al., 2021). On the other hand, these assays require a more demanding preparation and complex incubation environment and, thus, also bear a higher risk of infection and embryo mortality.

In angiogenesis research, the CAM assay has been broadly used to investigate basic mechanisms of blood vessel development, including the migration, proliferation and differentiation of endothelial cells (Ausprunk et al., 1974; Kurz et al., 1995) as well as ECM remodeling (Ausprunk, 1986). Moreover, it is well suited to study the pro- or anti-angiogenic effects of natural factors and pharmacological compounds (Jacoby et al., 2010; Farina et al., 2011). These can either be topically applied to the CAM (Liu et al., 2022) or administered systemically by intravascular injection (Storgard et al., 2005). In addition, the CAM frequently serves as host tissue to study the vascularization of implanted biomaterials as well as benign and malignant tissue grafts (Gescher et al., 2005; Ademi et al., 2021; Ara et al., 2022). The latter ones can be analyzed independently of their species origin without the risk of immunological rejection, because the early chicken embryo lacks a functional immune system (Baiguera et al., 2012). However, the analysis of their vascularization is only possible during a relatively short time period of ∼10 days, because chicken embryos already hatch on developmental day 21. Correspondingly, in most countries CAM-based studies are restricted to developmental day 14–15 to fulfil the criterion of animal-free experiments (Nowak-Sliwinska et al., 2018).

There are many possibilities to visualize and analyze blood vessel formation and patterning in the CAM assay. These range from simple macroscopic inspection, light and fluorescence microscopy to sophisticated imaging technologies, including ultrasonography, optical Doppler tomography, microcomputed tomography and magnetic resonance imaging (Nowak-Sliwinska et al., 2014; Moreno-Jiménez et al., 2017; Eckrich et al., 2020). Obviously, they markedly differ in many aspects, such as resolution, required equipment and expense. Hence, the choice of the right approach is crucially dependent on the research question and the present laboratory conditions. Furthermore, it should be considered that quantitative analyses in the CAM assay (e.g., the measurement of microvessel densities or branching points) are quite challenging, because of the high variability of the angiogenic response and embryo-induced movements of the CAM. Therefore, multiple measurements need to be performed to generate statistically valid data sets. This, however, is not a major problem considering the fact that chicken eggs are quite cheap and the technical preparation of the CAM is easily feasible. Thus, this versatile assay is also suitable for large-scale *in vivo* screenings.

### Zebrafish model

**FIGURE 4:**
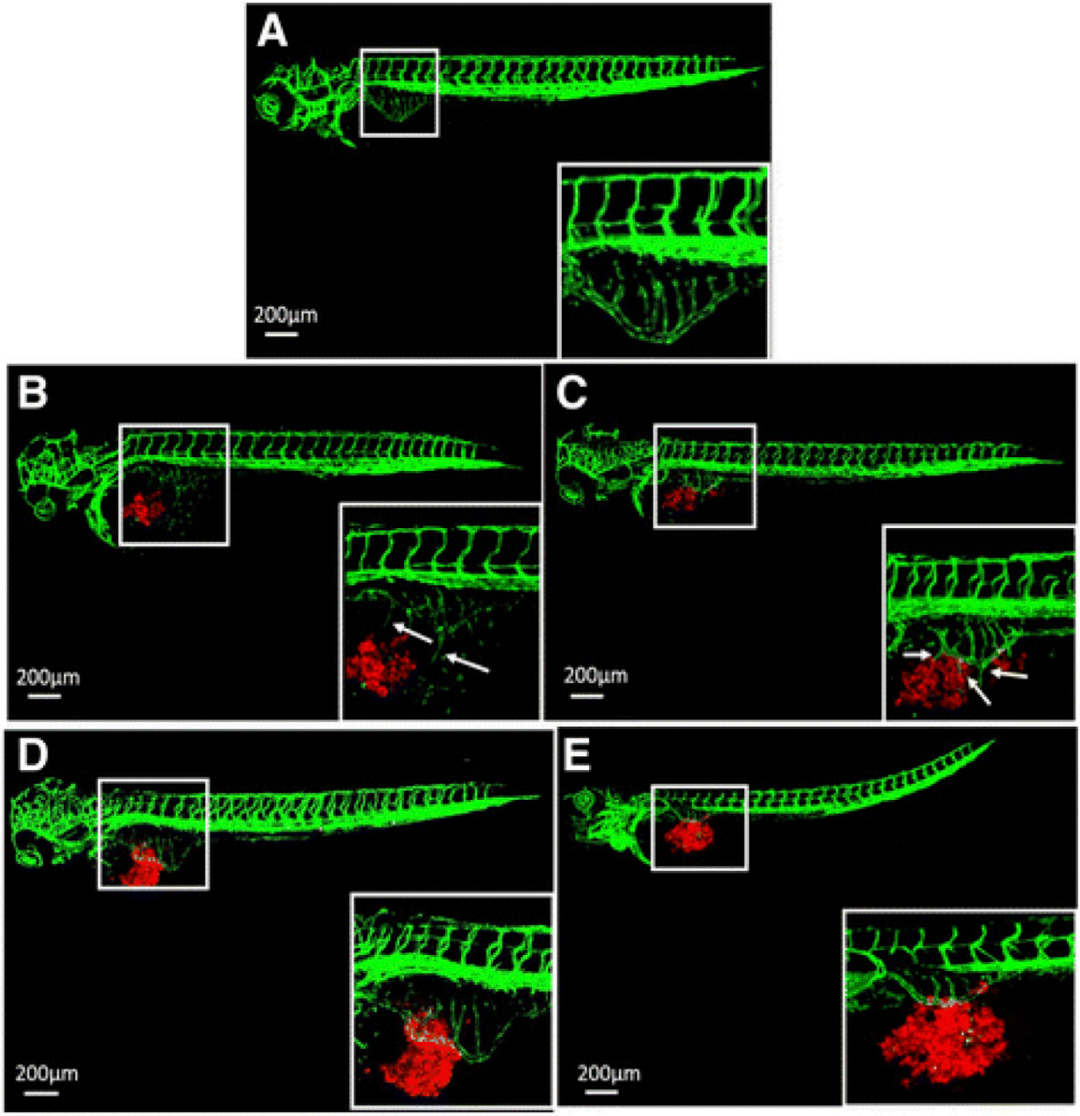
Inhibition of tumor angiogenesis in zebrafish larvae according to Wu et al. (2017). **(A)** Typical confocal microscopic images of subintestinal vessels of an uninjected transgenic *fli-eGFP* zebrafish larva at 3 days post fertilization. **(B,C)** Fluorescently labeled (CM-DiI) gastric cancer AGS cells **(B)** and SGC-7901 cells **(C)** were injected to zebrafish larvae and induced angiogenesis at day 1 post injection. **(D,E)** 50 nM VRI inhibited angiogenesis of the subintestinal vessels caused by the cell lines AGS **(D)** and SGC-7901 **(E)**. The white boxes at lower right corner show the higher magnification of the upper left white boxes. The arrows indicate the tumor cell-induced angiogenesis. Reproduced with permission from BioMed Central under the terms of the Creative Commons Attribution 4.0 International License (http://creativecommons.org/licenses/by/4.0/).

The zebrafish (*Danio rerio*) is a versatile and widely used *in vivo* model in angiogenesis research, which combines several essential advantages. This fish is easy to keep under laboratory conditions and produces hundreds of larvae per week through mating, which enables large-scale screenings (Lieschke and Currie, 2007). In early developmental stages younger than 120 h, these larvae lack the legal status of experimental animals, although they already exhibit a rudimentary yet functional cardiovascular system 24 h after fertilization (Isogai et al., 2001). Moreover, they are transparent, which provides easy access to their microcirculation and internal organs for microscopic *in vivo* imaging. Since ∼70% of the human genes have an orthologue in the zebrafish genome (Howe et al., 2013), the prediction quality of pharmacological testings in zebrafish larvae for human applications is good. In addition, the zebrafish is suitable for genetic manipulation. Accordingly, numerous transgenic zebrafish lines are available by now. They allow the visualization of endothelial cells and their precursors, perivascular cells and blood cells during vascular network development by means of cell-specific expression of fluorescent reporter proteins (Chávez et al., 2016). On the other hand, gene silencing and editing, which have originally been widely performed by the application of morpholino antisense oligonucleotides (Wyatt et al., 2015) and more recently by means of TALEN (Bedell et al., 2012) and CRISPR/Cas9 (Cornet et al., 2018), offer the opportunity to study the function of individual genes during vasculogenesis, sprouting angiogenesis and vascular remodeling (Eberlein et al., 2021). In this context, it should be mentioned that zebrafish larvae are able to survive without a functional vascular system by passive oxygen diffusion up to 5 days (Stainier et al., 1996). This also enables the investigation of late phenotypes of vascular malformations, which would be otherwise lethal in living mammals (Isogai et al., 2001; Peterson et al., 2004; Chávez et al., 2016). In addition, it is possible to inject human cells into the larvae without immunological rejection. This allows the analysis of angiogenesis in a humanized tissue-specific environment. For instance, Wu et al. (2017) demonstrated a potent anti-angiogenic effect of VRI, a pyridinyl-anthranilamide compound inhibiting the kinase activities of both VEGF receptor-1 and 2, on the xenografted fluorescently labeled gastric cancer cell lines AGS and SGC-7901 in transgenic *fli-eGFP* zebrafish embryos (Figure 4).

Despite all these advantages, it has to be considered that zebrafish larvae rapidly develop with major changes in their organ architecture and cardiovascular system over time. Accordingly, they are not suitable for long-term studies. Furthermore, they are aquatic organisms and, thus, markedly differ in many physiological aspects, such as their respiration, from mammals (Chávez et al., 2016). Therefore, promising results achieved by means of zebrafish larvae may need to be further validated in mammalian models, which represent closer substitutes for humans. Nonetheless, there is no doubt that the number of complex experiments in mammals can be markedly reduced by means of pharmacological and genetic screening studies in the zebrafish model.

## Mathematical modeling

**FIGURE 5:**
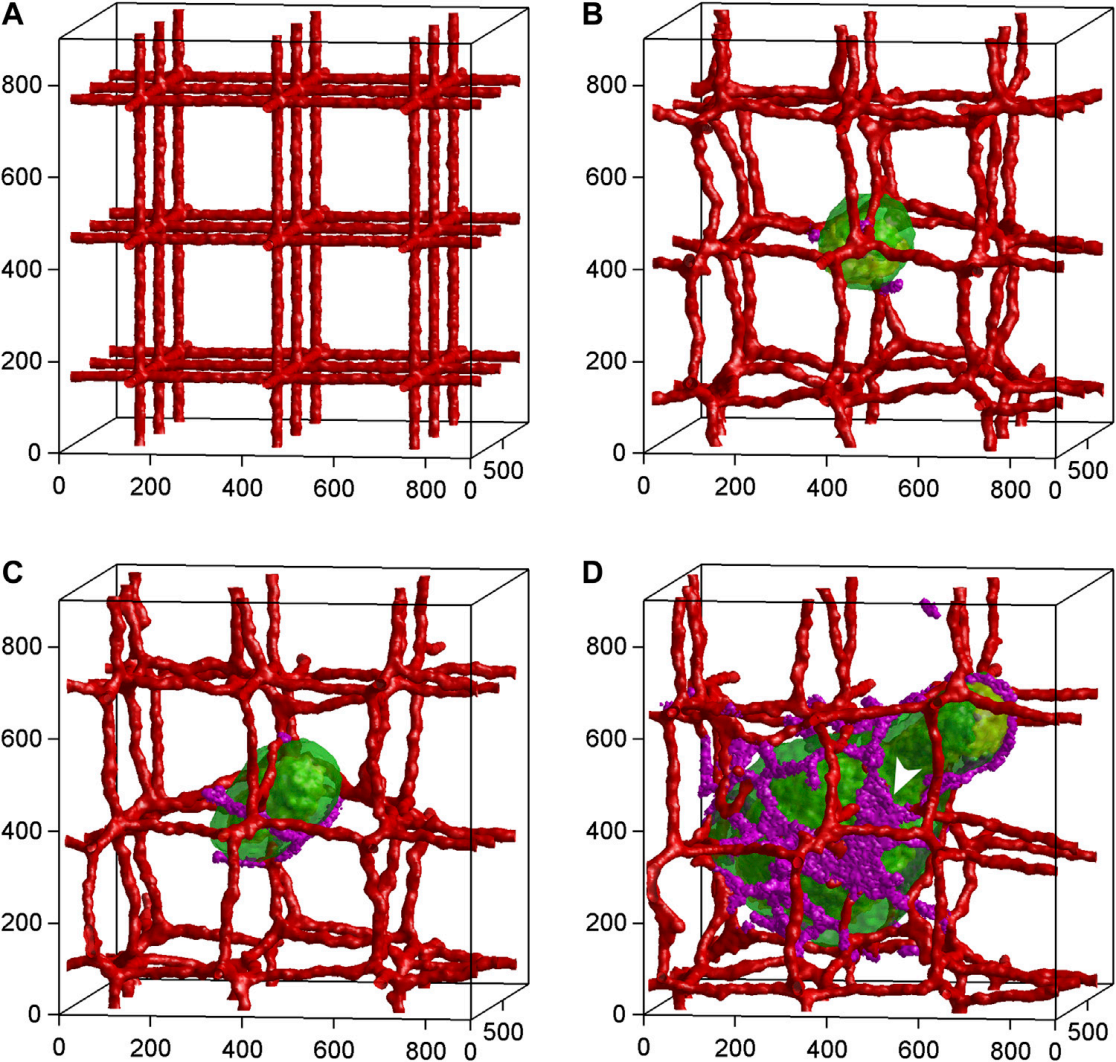
Time-series of 3D tumor growth and angiogenesis according to a mathematical model of Shirinifard et al. (2009). **(A)** Day 0: The pre-existing vasculature and the initial normal tumor cell. **(B)** Day 15: The tumor grows into a sphere with a maximum diameter of about 300 µm. The purple cells are active neovascular cells. **(C)** Day 30: The tumor grows into a cylinder with a length of about 350 µm and a diameter of about 300 µm. The vasculature is about to rupture. **(D)** Day 75: The developed vascularized tumor. The white arrowhead shows neovascular cells organized into 2D sheets. Cell types: Green: normal; yellow: hypoxic; red: vascular; purple: neovascular. Axes are labeled in µm. Reproduced with permission from PloS ONE under the terms of the Creative Commons Attribution 4.0 International License (http://creativecommons.org/licenses/by/4.0/).

Mathematical modeling combines experimental data and physical laws to simulate angiogenesis and tissue vascularization *in silico*. Particularly in cancer research, this approach has been continuously developed further since its initiation by Anderson and Chaplain (1998) to gain detailed information about tumor-driven blood vessel formation and remodeling as well as intra-tumoral oxygen, nutrient and drug distribution (Shirinifard et al., 2009; Welter and Rieger, 2016; Suzuki et al., 2018) (Figure 5). In this context, it allows to identify general biological principles of angiogenesis and to set up predictive models for the testing of anti-angiogenic therapeutic regimens (Venkatraman et al., 2016; Lai and Friedman, 2019; Akbarpour Ghazani et al., 2020; Mousavi et al., 2022). For this purpose, mathematical modeling of the tumor vasculature can be performed at the cell or the tissue scale by means of discrete (i.e., endothelial cells are treated as individual objects), continuous (i.e., endothelial cells are treated as concentrations) or hybrid (i.e., a combination of discrete and continuous approaches) models, as recently reviewed in detail by Hormuth et al. (2021). However, it should be noted that many parameters in these models are often assumed values (Pamuk et al., 2018), which may limit the biological relevance and predictive power of the generated results. Hence, it is necessary to continuously improve the calibration and validation of mathematical angiogenesis models by means of biologically based data. This can be achieved by time-resolved imaging and quantification of vascular dynamics under experimental *in vivo* conditions (Perfahl et al., 2011). Although animal models may provide such conditions, they are laborious and not suitable for high-throughput experiments. Accordingly, in the future they may be gradually replaced by microfluidic approaches, which allow the isolated analysis of specific mechanisms during blood vessel formation and drug testing in highly controlled, repeatable but complex experimental settings (Hormuth et al., 2021). This may also open the door for a broad implementation of computer simulations in combination with artificial intelligence into clinical practice and, thus, for the establishment of personalized therapeutic regimens with improved efficacy and less side effects (Bodzioch et al., 2021).

## Conclusion

The development of new blood vessels is a dynamic process, which is not only dependent on the coordinated interaction of endothelial and perivascular cells of the microvasculature, but also crucially determined by hemodynamic forces, the local tissue environment and systemic factors. *In vivo* angiogenesis studies in animal models are considered to reflect these complex conditions and, thus, to provide data of high physiological relevance for human applications. On the other hand, they are not suitable for large-scale screenings, because they are laborious and expensive. Most particularly, however, they confront researchers with serious ethical concerns. Indeed, in line with the 3R principle of Russell and Burch, it is the obligation of the scientific community to continuously establish and refine approaches, which enable an animal-free research of highest quality standards and relevance for basic science and clinical practice. In the present narrative review, we discuss such approaches without any claim to completeness, because we did not perform a systematic literature search. Accordingly, we are aware that we may have missed the one or other interesting assay or model. Nonetheless, we feel that the herein selected and discussed approaches provide an excellent blue print how an animal-free research can be achieved. In fact, they demonstrate that even complex processes, such as angiogenesis, can be analyzed in detail from various viewpoints to gain a valid overall picture. Currently, this usually implies the reasonable combination of different assays in pre-screening studies assessing the pro- or anti-angiogenic activity of test compounds. Such pre-screening studies already contribute to a drastic reduction of animal experiments in early stages of modern drug development. However, the future goal should be to analyze angiogenesis in all its complexity by means of physiologically relevant animal-free approaches without the additional need for a final validation of the results in an animal model. Rapid progress in the generation of tissue- and organ-mimicking microfluidic systems and mathematical modeling may pave the way to turn this fiction into reality.
